# Synergy and Mode of Action of Ceftazidime plus Quercetin or Luteolin on *Streptococcus pyogenes*

**DOI:** 10.1155/2015/759459

**Published:** 2015-10-21

**Authors:** Supatcharee Siriwong, Kanjana Thumanu, Tanaporn Hengpratom, Griangsak Eumkeb

**Affiliations:** ^1^School of Pharmacology, Institute of Science, Suranaree University of Technology, 111 University Avenue, Suranaree Subdistrict, Muang District, Nakhon Ratchasima 30000, Thailand; ^2^Synchrotron Light Research Institute (Public Organization), Suranaree Subdistrict, Muang District, Nakhon Ratchasima 30000, Thailand

## Abstract

*Streptococcus pyogenes* causes streptococcal toxic shock syndrome. The recommended therapy has been often failure through the interfering of beta-lactamase-producing bacteria (BLPB). The present study was to investigate antibacterial activity, synergy, and modes of action of luteolin and quercetin using alone and plus ceftazidime against *S. pyogenes*. The MICs of ceftazidime, luteolin, and quercetin against all *S. pyogenes* were 0.50, 128, and 128 *µ*g mL^−1^, respectively. A synergistic effect was exhibited on luteolin and quercetin plus ceftazidime against these strains at fractional inhibitory concentration indices 0.37 and 0.27, respectively, and was confirmed by the viable count. These combinations increased cytoplasmic membrane (CM) permeability, caused irregular cell shape, peptidoglycan, and CM damage, and decreased nucleic acid but increased proteins in bacterial cells. Enzyme assay demonstrated that these flavonoids had an inhibitory activity against *β*-lactamase. In summary, this study provides evidence that the inhibitory mode of action of luteolin and quercetin may be mediated via three mechanisms: (1) inhibiting of peptidoglycan synthesis, (2) increasing CM permeability, and (3) decreasing nucleic acid but increasing the protein contents of bacterial cells. So, luteolin and quercetin propose the high potential to develop adjunct to ceftazidime for the treatment of coexistence of the BLPB and *S. pyogenes* infections.

## 1. Introduction

**Figure 1:**
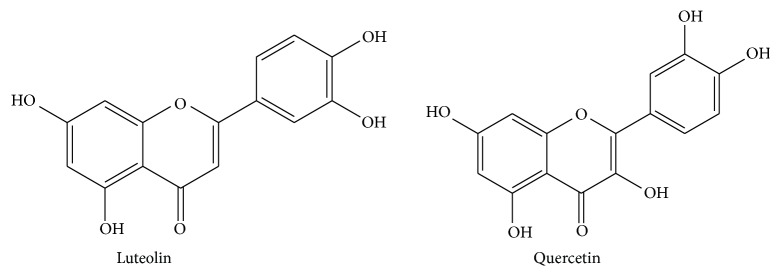
The chemical structure of luteolin and quercetin.

*Streptococcus pyogenes* (*S. pyogenes*; group A streptococcus) is an important species of gram-positive pathogens. It displays groups A antigen and beta-hemolysis. These strains are the most common cause of bacterial pharyngitis, scarlet fever, impetigo, puerperal sepsis, or childbed fever in the past and handle streptococcal toxic shock syndrome today. The group A streptococcus has been investigated for its significant role in the development of acute rheumatic fever, rheumatic heart disease, acute glomerulonephritis, and reactive arthritis [1, 2]. An increase in severe* S. pyogenes* diseases in the past two decades has been reported. The surveillance of severe* S. pyogenes* infection diagnosed during 2003 and 2004 in 11 countries across Europe showed that the risk of infection was highest among the elderly; skin and soft tissue were the most common of infections, 44% among patients who developed streptococcal toxic shock syndrome. These results confirm a high incidence of severe* S. pyogenes* disease in Europe [3]. Recommended therapies for* S. pyogenes* infections include penicillin and cephalosporin. However, resistance to drugs, via different mechanisms, has increased in* S. pyogenes* [4, 5]. Previous research found that* S. pyogenes* 1.9% was intermediately resistant to ampicillin, and 0.3% and 1.9% were resistant to chloramphenicol and azithromycin, respectively [6]. The coexistence of oropharyngeal BLPB may not have only survived penicillin therapy but could also have protected other penicillin-susceptible bacteria from penicillin. Thus, the increased failure rate of penicillin and cephalosporin in eradication of otitis, sinusitis, pharyngeal-tonsillitis, and streptococcal toxic shock syndrome infections of these bacteria, such bacteria as* Haemophilus influenza, Moraxella catarrhalis, Klebsiella pneumoniae*, and* Pseudomonas aeruginosa*, had been reported [7, 8]. Effective antibiotics available for the treatment of* S. pyogenes* and the coexistence of the BLPB infections, for example, penicillin and ceftazidime, are frequently associated with the failure of *β*-lactams and unwanted side effects [7, 9, 10]. The invention of new combination agent to treat these infections that can reduce adverse drug effect is urgently needed. Plant-derived flavonoids, which occur abundantly in our daily dietary intake, possess antitumor and antibacterial properties, which is one of the most interesting sources of new therapeutics. Previous findings reported that luteolin, that appeared nontoxic, and quercetin are effective antileishmanial agents and luteolin could be a strong candidate for antileishmanial drug design [11]. Besides, Chiruvella et al. found that luteolin-7-O-glucoside, ethyl acetate extract from* Soymida febrifuga* (Roxb.), had an antibacterial effect against* Bacillus subtilis* and* Salmonella typhimurium*, respectively [12]. In the same way, luteolin derivatives showed the most favorable antibacterial activity in vitro with MICs of 1.562, 3.125, 3.125, and 6.25 *μ*g mL^−1^ against* B. subtilis, S. aureus, P. fluorescens*, and* E. coli*, respectively [13]. Apart from this, Wang and Xie reported that luteolin showed clear antibacterial activity against* Staphylococcus aureus* by DNA topoisomerase I and II inhibition, which resulted in some decrease in the nucleic acid and protein synthesis [14]. Previous findings about quercetin, that has been found in onions, tomatoes, and honey, reported that it was proposed to inhibit gyrases through two different mechanisms based on interaction either with DNA or with ATP binding site of gyrase [15]. In the same way, quercetin showed potent antibacterial activity against a wide spectrum pathogen responsible for hospital-acquired and community-acquired by bacterial DNA gyrase and topoisomerase IV inhibition [16]. What is more, quercetin was fed to guinea pigs and it was found that it decreased* H. pylori* infection in the gastric mucosa and reduced both the inflammatory response and lipid peroxidation [17]. In addition, Li and Xu concluded that quercetin extracted from lotus leaves may have been a potential antibacterial agent for periodontitis [18]. Also, quercetin showed antibacterial activity against* Escherichia coli*,* Pseudomonas aeruginosa*,* Staphylococcus aureus*, and* Enterococcus faecalis* [19]. Besides, Hossion and Sasaki reported that novel quercetin glycoside showed antibacterial agents against vancomycin-resistant bacterial strains [20]. However, Razavi et al. found that quercetin 3-O-glucoside (Q3G) had no antibacterial effects and low cytotoxicity [21]. Many flavonoids isolated from plants have shown synergistic antibacterial activity [22]. For example, Ramos et al. discovered that quercetin derivatives, extracted from onion (*Allium cepa*) skin, showed antibacterial activity against MRSA and* H. pylori* strains and increased susceptibility of MRSA to *β*-lactams [23]. Furthermore, previous findings found that quercetin plus ceftazidime and luteolin plus amoxicillin exhibited synergistic activity against ceftazidime-resistant* S. aureus* and amoxicillin-resistant* E. coli*, respectively [22, 24]. Moreover, Gopu et al. revealed that quercetin could act as a competitive inhibitor for signaling compound towards the LasR receptor pathway and served as a novel QS-based antibacterial/antibiofilm drug to manage food-borne pathogens and its synergistic activity with conventional antibiotics could enhance the susceptibility of tested pathogens [25]. From these findings, the result of Q3G is still ambiguous. So, our studies needed to investigate the effect of quercetin and luteolin, which is abundant in our daily dietary intake on the* S. pyogenes.* Furthermore, no work has been done on the synergistic effect of ceftazidime plus either luteolin or quercetin on* S. pyogenes* and the coexistence of the BLPB strains. To this aim, the present study investigated antibacterial and synergistic activities of selected flavonoids, luteolin and quercetin (Figure 1), used either alone or in combination with ceftazidime against* S. pyogenes*. The elementary mechanism of action was also examined. Also, the effect of these agents on the changes of the biochemical component was investigated by FT-IR microspectroscopy [26, 27].

## 2. Material and Methods

### 2.1. Materials and Bacterial Strains

The* S. pyogenes* DMST 30653 (*S. pyogenes*), 30654, and 30655 were obtained from the Department of Medical Sciences, Ministry of Public Health, Thailand. The origin of these strains used in the study was obtained from inpatient in the infectious disease ward from twelve provincial hospitals in the North-Eastern area of Thailand. Each* S. pyogenes* strain used in this research was swabbed and isolated from only one anatomical site of each inpatient that was phlegm from the throat of the patient (*n* = 4). The* S. aureus* ATCC 29213, positive control, was purchased from American Type Culture Collection (ATCC), USA. Luteolin (purity 98%) and quercetin (purity 99%) were purchased from the Indofine Chemical Company (New Jersey, USA) (Figure 1). Ceftazidime, amoxicillin, penicillin, *β*-lactamase type IV, dimethyl sulfoxide (DMSO), glutaraldehyde (grade I, 25% for EM), osmium tetroxide (4% for EM), Spurr Low-Viscosity Embedding Kit, and nisin (from* Lactococcus lactis*, 2.5% balance sodium chloride and denatured milk solids) were obtained from Sigma (Sigma-Aldrich, UK). Mueller-Hinton agar (MHA), Mueller-Hinton agar with sheep blood (5% v/v) (MHA-SB), cation-adjusted Mueller-Hinton broth (CAMHB), and cation-adjusted Mueller-Hinton broth with lysed horse blood (2.5% v/v) (CAMHB-LHB) were obtained from Oxoid (Basingstoke, UK).

### 2.2. Bacterial Suspension Standard Curve

Bacterial suspensions standard curve method was performed to determine known viable count following the method of Richards and Xing with little modifications [28]. Briefly, to select bacterial suspensions with a known viable count the following steps were performed. A loopful of* S. pyogenes* and* S. aureus* was used to inoculate 100 mL quantities of the CAMHB-LHB. The cultures were incubated at 37°C for 20 h. The bacterial cells were pelleted by centrifuging at 6,000 ×g for 10 minutes (min). The cells were then washed two times by suspending and centrifuging at 6,000 ×g for 5 min in 10 mL 0.9% NaCl, resuspended in 50 mL sterile 0.9% NaCl, and diluted, so that 5-6 spectrophotometer readings could be obtained over the absorbance range of approximately 0.05–0.25 at a wavelength of 500 nm. For example, the following were selected: 0.05, 0.10, 0.15, 0.20, and 0.25. Viable counts for each absorbance reading were determined in triplicate using an overdried agar plate counting method.

### 2.3. MICs Determination

The MICs of ceftazidime, amoxicillin, penicillin, nisin, luteolin, and quercetin against* S. pyogenes* and* S. aureus* strains were performed following the method of those of Liu et al., Eumkeb et al., and Clinical and Laboratory Standards Institute [22, 29, 30]. Shortly, the suspension was adjusted to approximately 1 × 10^8^ CFU mL^−1^. Then, the suspension of 1 × 10^6^ CFU mL^−1^ was achieved from tenfold serial dilution. The final concentration approximately 1 × 10^5^ CFU mL^−1^ of testing bacteria in each antibacterial agent was accomplished by adding the 0.1 mL of diluted inoculum of each stain to 0.9 mL of CAMHB-LHB for* S. pyogenes* and CAMHB for* S. aureus* plus serial dilutions of the tested agents. Antibiotics used and flavonoids were prepared to obtain stock solutions at 1,024 *μ*g mL^−1^ by dissolving in sterile distilled water and 0.1% DMSO, respectively. The respective concentration was implemented by serially twofold dilution of the stock. The lowest concentration that showed no visible growth after incubating at 37°C for 20 h was reported as the MIC.* S. aureus* ATCC 29213 was used as a reference strain. The investigation was performed in three experimentations, each experiment was operated in triplicate, and data are shown as the mean of three experiments.

### 2.4. Checkerboard Determination

Checkerboard assay to determine the synergistic activity of flavonoids in combination with ceftazidime against* S. pyogenes* was executed following Eumkeb et al. and Sabath [22, 31]. To sum up briefly, the 0.25 mL of 5 × 10^6^ CFU mL^−1^ bacterial suspensions was added to a dilution 2.25 mL CAMHB-LHB plus 10% serial dilution of the flavonoids plus ceftazidime to give 5 × 10^5^ CFU mL^−1^. Tubes of the bacterial suspensions in broth without antibacterial agent were used as the control. The cultures were incubated for 20 h at 37°C. The tests were carried out in triplicate. The MICs were determined for each antibacterial combination and the isobolograms were plotted. The interaction between the two agents was calculated by the fractional inhibitory concentration (FIC) index of the combination. The FIC of each agent was calculated by the complete growth inhibition of microorganism in combination tube. The following formula was used for the FIC index (FICI) calculation: FIC of quercetin = MIC quercetin in the combination/MIC of quercetin alone; FIC of ceftazidime = MIC of ceftazidime in the combination/MIC of ceftazidime; so, FICI = FIC of quercetin + FIC of ceftazidime. In summation, the FIC index is determined by Marques et al. that when the FICI of the combination is less than or equal to 0.5, the combination is termed as synergistic; when FICI falls between greater than 0.5 and less than 1.0, it means partially synergistic; when FICI value is 1.0, it means additive; when FICI is between greater than 1.0 and less than 4.0, it means indifferent; and if FICI is greater than 4.0, it displays antagonistic activity between two compounds [32].* S. aureus* ATCC 29213 was used as a positive control. The FIC index is presented as the median values obtained from three independent experiments; each experiment was performed in triplicate.

### 2.5. Determination of Viability Curves

The killing curve determination was performed to confirm the synergistic activity of the combination following Richards and Xing, Eumkeb et al., and Clinical and Laboratory Standards Institute methods with slight modifications [22, 28, 30]. To summarize, after the FIC index was obtained, the MIC of each compound that gave synergism FIC index of the combination was chosen to investigate its mechanism of action. The half-MIC value of ceftazidime, luteolin, and quercetin alone and the MICs of these combinations that gave synergistic FIC index value were picked against* S. pyogenes* [33]. In brief, the viabilities of* S. pyogenes* at 5 × 10^5^ CFU mL^−1^ after exposure to these agents alone and in combination at nine distinct times (0, 0.5, 1, 2, 3, 4, 5, 6, and 24 h) were counted. Aliquots (0.1 mL) of each exposed time were transferred and diluted in 0.9% sodium chloride as needed to compute 30–300 colonies. The diluted cultures were dropped and spread thoroughly on plates containing MHA-SB. The growing colonies were counted after incubating at 37°C for 20 h. The lowest detectable limit for counting is 10^3^ CFU mL^−1^. Synergy was defined as a ≥ 100-fold or 2-log⁡10 decrease in colony count at 24 h by the combination compared with that by the most active single agent and as a ≥ 2-log⁡10 decrease in CFU mL^−1^ count compared with the starting inoculum. Additivity or indifference was defined as a < 10-fold change in colony count at 24 h by the combination compared with that by the most active single agent. If the increase in colony count ≥ 100-fold at 24 h by the combination compared with the most active drug alone, the antagonism was defined [32, 34, 35]. The experiment was performed in four observations, each observation was performed in triplicate, and data are shown as mean ± SEM.

### 2.6. The CM Permeability

The CM permeabilization experiment was executed as previously described by Shen et al. and Zhou et al. with some modifications [36, 37]. This method was performed by measurement of the release of UV-absorbing material concentrations using UV-VIS spectrophotometer. Briefly, subsequently the FIC index was obtained from checkerboard; the half-MIC value of ceftazidime, nisin, luteolin, and quercetin alone and the 3/4 MIC of these combinations that gave synergistic FIC indices were chosen against* S. pyogenes* to investigate the CM permeabilization. Nisin was used as a positive control [36]. High-performance liquid chromatography (HPLC) was used to measure the stability of benzylpenicillin to *β*-lactamase in the presence of an enzyme inhibitor. The quercetin and luteolin were preincubated with the enzyme at 37°C for 5 min prior to substrate addition. Reaction samples were injected at various times to Waters Bio-Sil C18 HL 90-5s reverse-phase column. Time-course assays were carried out using methanol/acetic acid (100 : 1) as stopping reagent. The analyses of the remaining substrate were determined by reverse-phase HPLC using acetonitrile/ammonium acetate as a mobile phase [24]. The research was examined in three studies, each study was operated in triplicate, and the graphs are displayed as mean ± SEM.

### 2.7. Enzyme Assay

The *β*-lactamase type IV of* Enterobacter cloacae* inhibition activity was previously described by Reading and Farmer with little modifications. The half-MIC concentrations of ceftazidime, luteolin, and quercetin alone were determined against the *β*-lactamase activity. Shortly, benzylpenicillin, a substrate for *β*-lactamase type IV, was calibrated to concentrations sufficient to hydrolyze 50–60% substrate within 5 min. The half-MIC of testing agents was preincubated with an enzyme in 50 mM sodium phosphate buffer (pH 7.0) at 37°C for 5 min prior to adding a substrate. The measured time at 0, 5, 10, 15, and 20 min was examined using methanol/acetic acid (100 : 1) as a stopping agent. Each sample at 10 *μ*L was injected to reverse-phase HPLC to analyze the remaining benzylpenicillin. The ammonium acetate (pH 4.5 acetic acid): acetonitrile (75 : 25) at 10 mM was injected as a mobile phase with flow rate 1 mL/min, UV detector at 200 nm, Ascentis C18 column, and 35°C for column temperature. The quantity of remaining benzylpenicillin was calculated by comparing the area under the chromatographic curve [24, 38]. The study was performed in three examinations; each examination was carried out in triplicate, and the graphs are displayed as mean ± SEM.

### 2.8. Transmission Electron Microscopy (TEM)

Cellular damage of bacteria was examined using TEM. Ceftazidime plus luteolin or quercetin that dramatically decreased the MICs against* S. pyogenes* was chosen for electron microscopy study when used singly and in combination. The subculture of this strain was prepared to be examined by TEM following Eumkeb et al. and Richards et al. with a minor modification [22, 39]. Concisely, after the FIC index was obtained from checkerboard, the half-MIC value of ceftazidime, luteolin, and quercetin alone and the 3/4 MIC of these combinations that gave synergistic FIC indices were picked against* S. pyogenes* to be investigated by TEM. This strain was preincubated at 37°C for 20 h; it was adjusted spectrophotometrically to obtain a final concentration approximately 5 × 10^5^ CFU mL^−1^. The cultures were grown in the tested agents at the concentrations as mentioned above, for 4 h with shaking 110 oscillations/min in a water bath at 37°C. Next, the cultures were harvested by centrifugation at 6,000 ×g for 15 min at 4°C, and the pellets were fixed in 2.5% glutaraldehyde (Electron Microscope Sciences; EMS) in 0.1 M phosphate buffer (pH 7.2) for 12 h. These cells were then meticulously washed twice with 0.1 M phosphate buffer. Then, the 1% osmium tetroxide (EMS) in 0.1 M phosphate buffer (pH 7.2) was added to the samples and left for 2 h at room temperature for postfixation. The samples were then washed in the buffer and gently dehydrated with graded ethanol (20%, 40%, 60%, 80%, and 100%, resp.) for 15 min. Afterwards, impregnation and embedding were performed using a degree of propylene oxide (EPP) : araldite and Spur's resin (EMS), respectively. An ultramicrotome with a diamond knife was applied to section these embedded samples and then mounted on copper grids. The ultrathin sections were then stained with 2% (w/v) uranyl acetate for 30 min and then 0.25% (w/v) lead citrate for 7 min. Lastly, these stained grids were investigated in a Tecnai G2 electron microscope (FEI, USA), operating at 120 kV. Furthermore, in order to confirm the effects of these tested agents either used singly or in combination on cell size, the cell area of these cells from micrographs was calculated by measuring cell width multiplied by cell length (nm^2^). The experiment was performed in three demonstrations; each demonstration was performed in triplicate, and the cell areas are shown as mean ± SEM.

### 2.9. The FT-IR Microspectroscopy Measurement

#### 2.9.1. Cell Preparation

Bacterial suspensions were exposed to the ceftazidime either singly or in combination with flavonoids and incubated temperature at 37°C for 24 h. Shortly, after the FIC index was elucidated from checkerboard, the half-MIC value of ceftazidime alone and the 3/4 MIC of these combinations that gave synergistic FIC indices were selected against* S. pyogenes* to perform the FT-IR investigation. Bacterial cells were prepared following the methods of Reading and Farmer, Eboigbodin and Biggs, and Eumkeb et al. with little modifications [24, 40, 41]. Briefly, these cells were incubated at 37°C in shaking water bath for four hours. The cell pellets were centrifuged at 3,000 ×g for 10 min and washed twice with saline. These cells were then washed twice with MilliQ water. A small portion of the pellet was then deposited on MirrIR low e-microscope slides (Kevey slide) to be used as a substrate for FT-IR microscope analysis. These cells were then desiccated under vacuum for about 20 min and stored in desiccators to form films suitable before analysis. To achieve high S/N ratios, 64 scans coadded were collected for each measurement in the wavenumber between 4,000 and 400 cm^−1^ resolution of 6 cm^−1^.

#### 2.9.2. Data Analysis

Spectra were recorded in reflection mode on a Bruker IR spectrometer (tensor 27) coupled to an IR microscope (Hyperion 2000) with 36x magnification. The data of the effect of variation of the composition and distribution of the biochemical components in bacterial cells during cell culture were analyzed using principal component analysis (PCA). All data analysis was carried out in the spectral range from 3000–2800 cm^−1^ to 1800–850 cm^−1^, which covers the fingerprint region [42–45]. The average peak area and intensity at each region were obtained from three independent examinations; each examination was done in triplicate.

### 2.10. Statistical Analysis

The experiment was performed in at least three experiments; each experimentation was performed in triplicate. All graph data are expressed as mean ± standard error of the mean (±SEM) due to the fact that it takes into account sample size. Significant differences of cell area in each treated group from TEM, CM permeability, and enzyme assay among each treated group at the same interval times and peak area in each group of FT-IR results were analyzed by one-way ANOVA. A *P* value < 0.01 of Scheffe's post hoc test was considered as a statistically significant difference.

## 3. Results

### 3.1. MICs and Checkerboard Determinations

The MICs of testing ceftazidime, nisin, luteolin, and quercetin against* S. pyogenes* are shown in Table 1. The results revealed that MICs of ceftazidime, nisin, luteolin, and quercetin against these strains were 0.50, 1, 128, and 128 *μ*g mL^−1^, respectively. These findings provide evidence that these* S. pyogenes* are still susceptible to ceftazidime [30]. The sensitive strain* S. aureus* ATCC 29213 was used as a positive control and was also susceptible to ceftazidime, amoxicillin, and penicillin (Table 1) [15]. Luteolin and quercetin exhibited little inhibitory effect against these strains. The FIC indices of ceftazidime plus luteolin or quercetin against all* S. pyogenes* strains were 0.37 or 0.27, respectively. From these results, these combinations showed synergistic activity against these strains following the description of Marques et al. as above mentioned (checkerboard determination) [32].

### 3.2. Killing Curve Determinations

**Figure 2:**
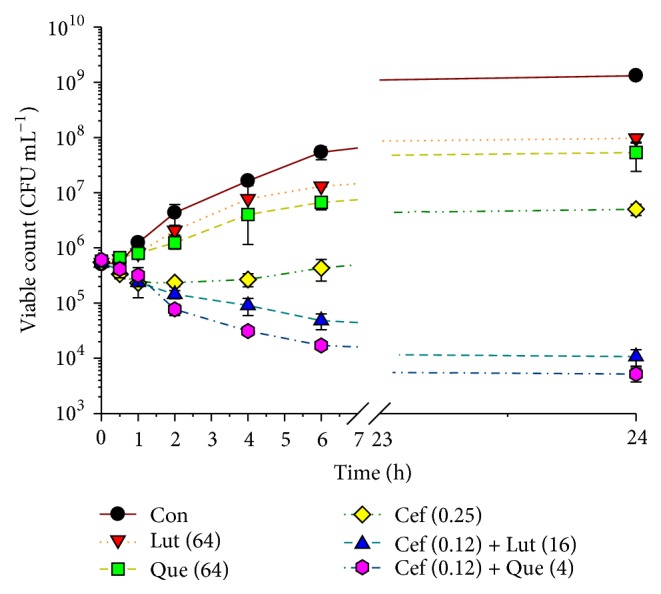
The effect of ceftazidime, luteolin, and quercetin either alone or in combination on the viable counts of* S. pyogenes* DMST 30653. Con = control (drugs free); Lut (64) = 64 *μ*g mL^−1^ luteolin; Que (64) = 64 *μ*g mL^−1^ quercetin; Cef (0.25) = 0.25 *μ*g mL^−1^ ceftazidime; Cef (0.12) + Lut (16) = ceftazidime 0.12 *μ*g mL^−1^ plus luteolin 16 *μ*g mL^−1^; Cef (0.12) + Que (4) = ceftazidime 0.12 *μ*g mL^−1^ plus quercetin 4 *μ*g mL^−1^. The experiment was performed in four observations and all graphs are shown as mean ± SEM.

The effects of ceftazidime, luteolin, and quercetin either alone or in combination on viable counts of* S. pyogenes* are revealed in Figure 2. The viable count of the cells treated with ceftazidime 0.25 *μ*g mL^−1^ alone was slightly lower than luteolin or quercetin at 64 *μ*g mL^−1^ alone between 2 and 24 h. Obviously, the combination of ceftazidime at 0.12 *μ*g mL^−1^ plus luteolin 16 *μ*g mL^−1^ or quercetin at 4 *μ*g mL^−1^ dramatically decreased the cells to 2.5 × 10^4^ and 6 × 10^3^ CFU mL^−1^, respectively, after 6 h and maintained the cells count at this level throughout 24 h. These results had also confirmed checkerboard determinations that ceftazidime plus luteolin or quercetin showed synergistic activity due to bacterial cells treated with these combinations was decreased ≥ 2-log⁡10 CFU mL^−1^ compared to ceftazidime alone treatment [35].

### 3.3. The CM Permeability Assay

**Figure 3:**
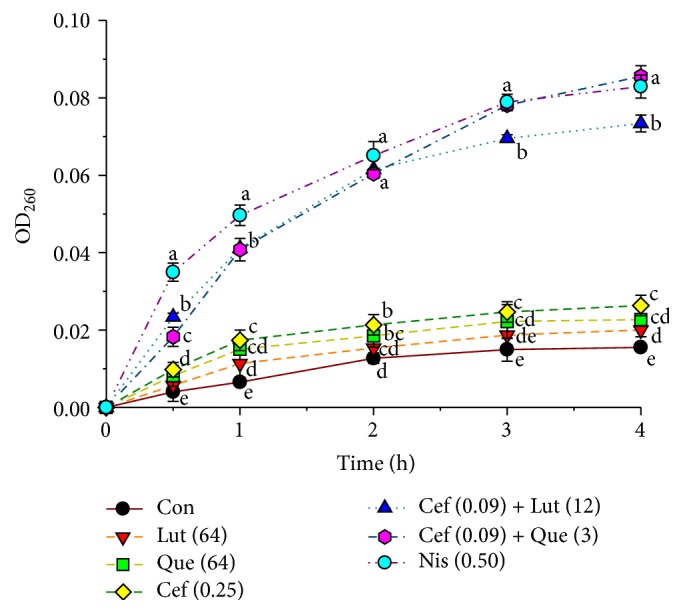
The presence of 260 nm absorbing the material in the supernatants of* S. pyogenes* DMST 30653 treated with luteolin, quercetin, and ceftazidime either alone or in combination. Con = control (drugs free); Lut (64) = 64 *μ*g mL^−1^ luteolin; Que (64) = 64 *μ*g mL^−1^ quercetin; Cef (0.25) = 0.25 *μ*g mL^−1^ ceftazidime; Cef (0.09) + Lut (12) = ceftazidime 0.09 *μ*g mL^−1^ plus luteolin 12 *μ*g mL^−1^; Cef (0.09) + Que (3) = ceftazidime 0.09 *μ*g mL^−1^ plus quercetin 3 *μ*g mL^−1^; Nis (0.5) = 0.5 *μ*g mL^−1^. Nisin at 0.5 *μ*g mL^−1^ was used as positive control, and untreated cells were used as negative control. The study was operated in three experiments, and all graphs are shown as mean ± SEM. Means sharing the same superscript are not significantly different from each other (Scheffe's, *P* < 0.01).

The CM permeability was measured by UV-absorbing release materials as presented in Figure 3. After treatment* S. pyogenes* cells with 0.50 *μ*g mL^−1^ nisin, 0.25 *μ*g mL^−1^ ceftazidime, ceftazidime at 0.09 *μ*g mL^−1^ plus luteolin at 12 *μ*g mL^−1^, and ceftazidime at 0.09 *μ*g mL^−1^ plus quercetin at 3 *μ*g mL^−1^ combination could induce the release of 260 nm absorbing material, which can be interpreted that mostly DNA, RNA, metabolites, and ions were significantly higher than controls, and luteolin and quercetin alone start from 0.5 h and throughout the 4 h (*P* < 0.01). The significant difference in increase in CM permeability strength in order at 4 h was ceftazidime plus quercetin > nisin > ceftazidime plus luteolin > ceftazidime ≥ quercetin ≥ luteolin > control (*P* < 0.01), respectively. These results imply that the synergistic activity of ceftazidime plus luteolin or quercetin increases cytoplasmic membrane permeability of this strain [36, 37].

### 3.4. Enzyme Assay

**Figure 4:**
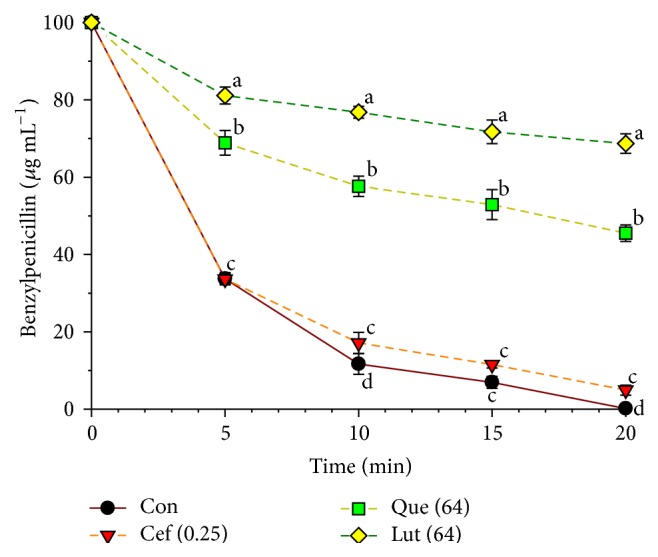
The inhibitory activity of luteolin, quercetin, and ceftazidime against *β*-lactamase in hydrolyzing benzylpenicillin. *β*-lactamase used from* E. cloacae*; Con = control (no testing agent), Cef (0.25) = 0.25 *μ*g mL^−1^, Que (64) = quercetin 64 *μ*g mL^−1^, and Lut (64) = luteolin 64 *μ*g mL^−1^. The graph shows the remaining benzylpenicillin at the same time. The research was executed in three studies, and all graphs are displayed as mean ± SEM. Means sharing the same superscript are not significantly different from each other (Scheffe's, *P* < 0.01).

The ability of luteolin and quercetin to inhibit the activity of *β*-lactamase type IV isolated from* E. cloacae* was assayed by determining the amount of remaining benzylpenicillin using reverse-phase HPLC. As shown in Figure 4, the result displayed that benzylpenicillin treated with luteolin, quercetin was significantly higher than control starting from 5 min (*P* < 0.01). The significant level of benzylpenicillin remainder from higher to lower was luteolin > quercetin > ceftazidime > control starting from 10 min and throughout the 20 min (*P* < 0.01). So, these findings provide evidence that luteolin and quercetin in combination with beta-lactam antibiotic may be useful to inhibit mixed BLBP bacteria and* S. pyogenes* in oropharyngeal infections [46, 47].

### 3.5. TEM

**Figure 5:**
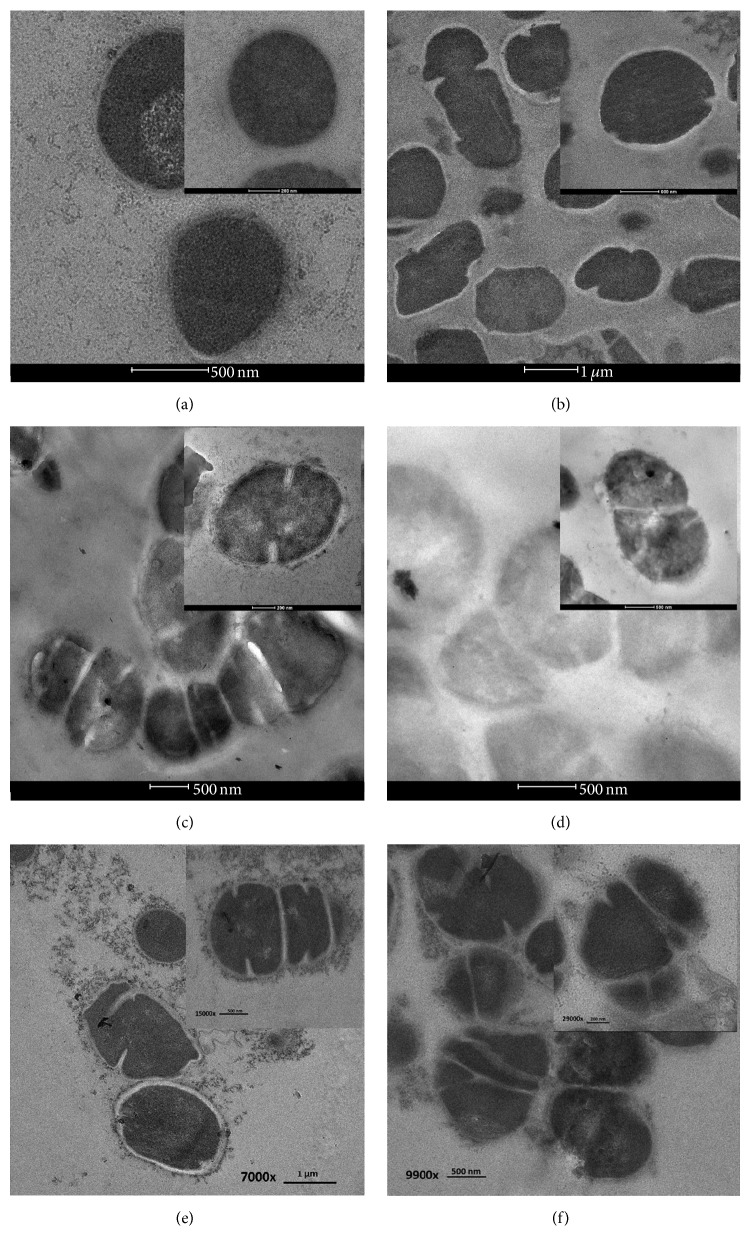
Ultrathin sections of log phase* S. pyogenes* DMST 30653 grown in CAMHB-LHB containing (a) control (drug-free); (b) ceftazidime (0.25 *μ*g mL^−1^); (c) luteolin (64 *μ*g mL^−1^); (d) quercetin (64 *μ*g mL^−1^); (e) ceftazidime (0.09 *μ*g mL^−1^) plus luteolin (12 *μ*g mL^−1^); (f) ceftazidime (0.09 *μ*g mL^−1^) plus quercetin (3 *μ*g mL^−1^). ((a) 195,000x, bar 500 nm; (b) 7,000x, bar 1 *μ*m; (c) 9,900x, bar 500 nm; (d) 15,000x, bar 500 nm; (e) 7,000x, bar 1 *μ*m; (f) 9,900x, bar 500 nm;* inset*: (a) 34,000x, bar 200 nm; (b) 17,000x, bar 500 nm; (c) 29,000x, bar 200 nm; (d) 15,000x, bar 500 nm; (e) 15,000x, bar 500 nm; (f) 29,000x, bar 200 nm).

**Figure 6:**
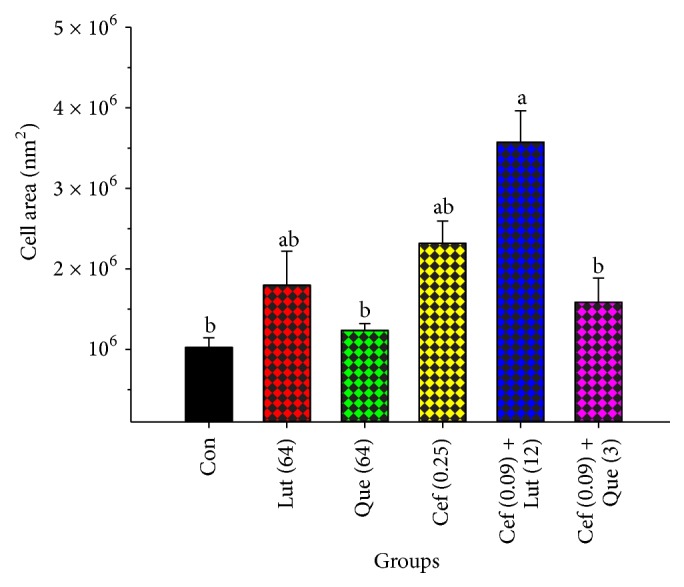
The effect of ceftazidime, luteolin, and quercetin alone and in combination on average cross section of* S. pyogenes* DMST 30653 cell areas from TEM. Con = control (drugs free); Lut (64) = 64 *μ*g mL^−1^ luteolin; Que (64) = 64 *μ*g mL^−1^ quercetin; Cef (0.25) = 0.25 *μ*g mL^−1^ ceftazidime; Cef (0.09) + Lut (12) = ceftazidime 0.09 *μ*g mL^−1^ plus luteolin 12 *μ*g mL^−1^; Cef (0.09) + Que (3) = ceftazidime 0.09 *μ*g mL^−1^ plus quercetin 3 *μ*g mL^−1^. The examination was carried out in three experiments and all graphs are illustrated as mean ± SEM. Means sharing the same superscript are not significantly different from each other (Scheffe's, *P* < 0.01).

Electron micrographs of log phase of* S. pyogenes* cells in the presence of luteolin, quercetin, and ceftazidime alone and ceftazidime plus luteolin or quercetin are presented in Figure 5. The peptidoglycan and cytoplasmic membrane can be distinguished from the control group. The morphology of the cells had normal appearance (Figure 5(a)). The* S. pyogenes* cells treated with ceftazidime alone are revealed in Figure 5(b). The cell division of a lot of these cells may be interrupted and delayed result in cell shape distortions. The average cross-sectional cell areas of these cells were larger than the control, but not significantly (*P* > 0.01) (Figure 6). The luteolin treated alone displayed a little repaired cytoplasmic membrane, and cell shape distortion or broken cells in a lot of these cells compared to the control (Figure 5(c)). The quercetin treated cells alone are presented in Figure 5(d). These treated cells revealed thinner or disappearing peptidoglycan, cytoplasmic membrane damage, cell shape distortion, and broken cells in many of these cells compared with controls. The average cell areas of these flavonoids treated alone were a bit larger than the control, despite not significantly different (*P* > 0.01). Furthermore, the micrograph of these cells after exposure to ceftazidime plus luteolin is shown in Figure 5(e). The result exhibited that the cell division of many of these cells may be interrupted leading to twisted and irregular cell shape. Some of these cells revealed peptidoglycan and cytoplasmic membrane damage. Obviously, the average cell areas of these cells were significantly larger than controls (*P* < 0.01) (Figure 6). Likewise, Figure 5(f) reveals the ceftazidime plus quercetin treated cells. The cell division process in most of these cells may also be interrupted resulting in deformed and eccentric cell shape. The peptidoglycan and the cytoplasmic membrane in most of these cells were damaged. These average cell areas were larger than the control, but not significantly (*P* > 0.01) (Figure 6).

### 3.6. FT-IR Spectroscopy Measurement

**Figure 7:**
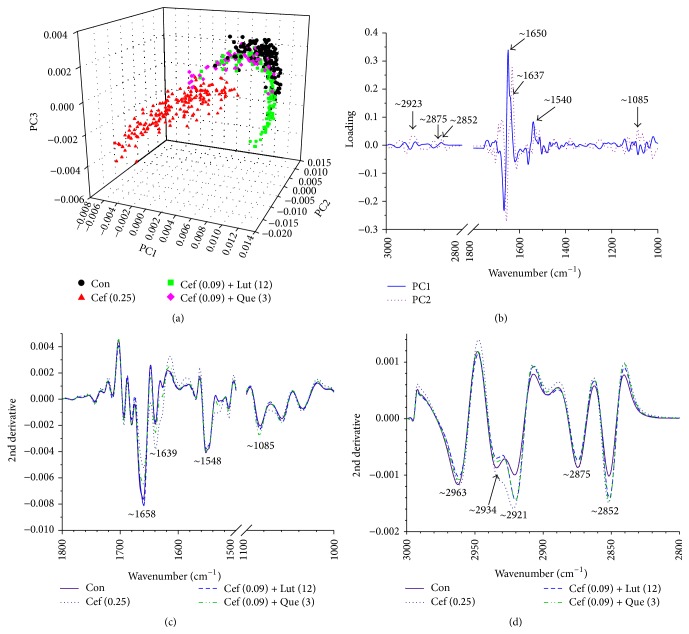
PCA results (a), the loading plot of the 1st (PC1) and the 2nd (PC2) principal components obtained from PCA of* S. pyogenes* DMST 30653 (b), the representative 2nd derivative transformation spectra (1800–1000 cm^−1^) (c), and the representative 2nd derivative transformation spectra (3000–2800 cm^−1^) (d). Symbols represent the FT-IR spectra of Con = control (drugs free); Cef (0.25) = 0.25 *μ*g mL^−1^ ceftazidime; Cef (0.09) + Lut (12) = ceftazidime 0.09 *μ*g mL^−1^ plus luteolin 12 *μ*g mL^−1^; Cef (0.09) + Que (3) = ceftazidime 0.09 *μ*g mL^−1^ plus quercetin 3 *μ*g mL^−1^. The investigation was performed in three studies and all data are displayed as the mean of three experiments.

The* S. pyogenes* strain was grown in CAMHB-LHB medium in the presence of 0.25 *μ*g mL^−1^ ceftazidime, ceftazidime at 0.09 *μ*g mL^−1^ plus luteolin at 12 *μ*g mL^−1^, and ceftazidime at 0.09 *μ*g mL^−1^ plus quercetin at 3 *μ*g mL^−1^ combination (3/4 FIC) and examined by FT-IR microspectroscopy. The loading plots are presented in Figure 7(b). The 1st loading displays 3 region coefficients at ~1650 cm^−1^, ~1637 cm^−1^, and ~1540 cm^−1^ (Figure 7(b)). These regions relate to average bands that are shown in Figure 7(c). The average peak areas and intensity at ~1658 and ~1639 cm^−1^ of these treated cells from higher to lower were ceftazidime plus luteolin > ceftazidime plus quercetin > ceftazidime > control which correspond with an absorption peak of secondary structure of protein amide I (alpha-helix and beta-sheet, resp.). Besides, the higher to lower average peak areas and intensity at ~1085 cm^−1^ were ceftazidime > control > ceftazidime plus quercetin > ceftazidime plus luteolin that correlate with an absorption peak of the phosphodiester backbone of nucleic acid (DNA and RNA) [48, 49].

The 2nd loading of these treated and control groups indicated that obvious regions at 3000–2800 cm^−1^ (~2934, ~2923, ~2875, and ~2852 cm^−1^) were corresponding to stretching mode of CH_2_ and CH_3_ in fatty acids of the various membrane amphiphiles and ester band, respectively (Figure 7(b)) [45, 50]. Obviously, these treated cells exhibited an average peak area and intensity of these peaks from higher to lower as ceftazidime plus luteolin > ceftazidime > control > ceftazidime plus quercetin (Figure 7(d)).

The PCA can be explained by the primary source of variation in the fingerprint region to differentiate and classify biomolecule of bacterial envelopes after treatment with these agents [51]. The 3-dimensional PCA clustering resulting from FT-IR spectral data of* S. pyogenes* after treatment with ceftazidime alone and combined with luteolin or quercetin is displayed in Figure 7(a). The biomolecular fingerprint clusters between controls, ceftazidime either alone or in combination with luteolin or quercetin groups, were clearly differentiated.

The loading from PC1 of* S. pyogenes* cells after treatment with ceftazidime either alone or in combination with luteolin or quercetin accounted for 75% of the total variability (PC1 55% and PC2 20%) and case of treating group loading PC2 accounted for 66% of the total variability (PC2 57% and PC3 9%) (Figure 7(a)).

## 4. Discussion

Flavonoids have inhibitory activity against a variety of bacteria. Many researchers described that flavonoids, including quercetin and various quercetin glycosides, possessed antibacterial activity [52, 53]. The MIC results revealed that* S. pyogenes* were still susceptible to ceftazidime alone because the standard value of the susceptibility of this drug against this strain is 0.50–4.0 *μ*g mL^−1^ [30]. Moreover, both luteolin and quercetin demonstrated little bacteriostatic effect against these strains with MIC 128 *μ*g mL^−1^. In addition, the MICs of quercetin and amoxicillin against penicillin-resistant* S. aureus* strains were > 400 and 250 *μ*g mL^−1^, respectively [22]. These MIC results are in substantial agreement with previous research that luteolin showed a higher activity against* E. coli* ATCC 8739 and* E. coli* DMST 20662 at MICs for 125 and > 200 *μ*g mL^−1^, respectively [24, 54].

The checkerboard determination revealed synergistic effects of ceftazidime plus luteolin or quercetin against* S. pyogenes* with FIC indexes at 0.37 and 0.27, respectively. These results are in correspondence with previous findings that quercetin plus conventional antibiotics and quercetin derivatives plus *β*-lactams enhanced the susceptibility of food-borne pathogens and MRSA, respectively [23, 25]. In addition, these findings are consistent with those of Eumkeb et al. that quercetin plus amoxicillin exhibited synergistic activity against penicillin-resistant* S. aureus* strains at FIC indices <0.05 [22]. In the same way, previous studies reported that a synergistic effect between quercetin and oxacillin against vancomycin-intermediate* S. aureus* displayed the lowest FIC index value of 0.0417 [55]. Similarly, these results are consistent with those of Eumkeb et al. that luteolin plus ceftazidime revealed synergistic effect against* E. coli* DMST 20662 at FIC index < 0.47 [24].

The killing curve results also confirmed the synergistic effect of ceftazidime plus luteolin or quercetin by reduction of ≥ 2-log⁡10 CFU mL^−1^ compared to ceftazidime alone treatment.

The CM permeability exhibited that luteolin and quercetin alone slightly increased CM permeability of this strain. Similarly, the combination of these flavonoids and ceftazidime significantly dramatically increased CM permeability compared to controls (*P* < 0.01). Obviously, the ceftazidime plus quercetin displayed higher CM permeability than nisin, a positive control, but not a significant difference at four hours (*P* > 0.01). These results are in substantial agreement with previous findings that luteolin either alone or combined with amoxicillin and apigenin alone or plus ceftazidime increased CM permeability of amoxicillin-resistant* E. coli* and ceftazidime-resistant* E. cloacae*, respectively [24, 56]. The increase in CM permeability may be one of the synergistic actions of these combinations against* S. pyogenes.* These results can be explained by assuming that the phospholipids bilayer in the plasma membrane might be damaged resulting in leaked cytoplasmic membrane [37].

The result of enzyme assay found that luteolin and quercetin had an inhibitory activity against *β*-lactamase type IV from* E. cloacae*. Clearly, these findings seem consistent with previous findings that galangin and kaempferide showed marked inhibitory activity against penicillinase (*β*-lactamase) type IV from* E. cloacae* [22, 43]. However, whether* S. pyogenes* produces beta-lactamase or not, previous study exhibited that the beta-lactamase produced by other bacteria in the pharynx could potentially inactivate the penicillin, resulting in increased treatment failures or infection relapses [57]. Besides, additional previous research revealed that amoxicillin alone therapy failed to eliminate* S. pyogenes* from a wound infection in the presence of a beta-lactamase-producing strain of* S. aureus* and suggested the potential of beta-lactamase inhibitor combination in the treatment of mixed bacterial skin infections involving beta-lactamase-producing organisms [46]. Moreover, BLPB may not have only survived penicillin therapy but can also protect other penicillin-susceptible bacteria from penicillin by releasing the free enzyme into their environment [47]. So, these findings provide evidence that luteolin and quercetin in combination with ceftazidime may be useful to inhibit mixed BLBP and* S. pyogenes* in oropharyngeal infections.

TEM results of* S. pyogenes* cells after exposure to ceftazidime plus luteolin or quercetin exhibited that cell division of many cells may have been interrupted leading to twisted and irregular cell shape and revealing peptidoglycan and CM damage. Clearly, the average cell areas of these cells were larger than controls. These results seem consistent with previous findings that the combination of ceftazidime plus galangin caused damage to the ultrastructures of the cells, affected the integrity of the cell walls, and led to an increase in cell size of ceftazidime-resistant* S. aureus* [22]. These results can be explained by assuming that luteolin and quercetin may insert synergistic action with ceftazidime to inhibit peptidoglycan synthesis and CM damage leads to marked morphological damage and delay cell division.

In general, previous findings revealed that the bactericidal effect of chlorine caused changes in the second derivative ATR spectra because of alteration in bacterial ester functional groups of lipids, structural proteins, and injured bacterial cells [26]. Our FT-IR results exhibited that fatty acids of* S. pyogenes* cells treated with quercetin plus ceftazidime and luteolin plus ceftazidime were decreased and increased, respectively, compared to controls. The nucleic acid of these combination treated cells was decreased, but amide I of proteins was increased compared to control [49]. Interestingly, the effects of luteolin on gram-positive* S. pyogenes* of these findings are perhaps similar to the effects on gram-negative, amoxicillin-resistant* E. coli* of previous findings that luteolin either alone or combined with amoxicillin caused an increase in fatty acids compared with control [24]. These results lead us to believe that luteolin or quercetin in combination with ceftazidime may affect the content of fatty acid chains on the various membrane amphiphiles resulting in cytoplasmic membrane damage, increase in cytoplasmic membrane permeabilization, and releasing nucleic acid from the cells. Also, the protein structures of these treated cells were shifted between amide I of *α*-helical structures and *β*-pleated sheet and DNA topoisomerases I, II, DNA gyrase, and topoisomerase IV could have been inhibited by these flavonoids resulting in protein accumulation [14–16].

## 5. Conclusions

In summary, our study provides evidence that luteolin and quercetin have the synergistic effect with ceftazidime against* S. pyogenes* and *β*-lactamase. Three modes of actions would be implying that these combinations inhibit peptidoglycan synthesis and decrease nucleic acid but increase amide I of proteins in bacterial cells and increase CM permeability. Naturally, luteolin and quercetin have restricted, limited toxicity. So, these flavonoids are proposed potentially to be used as an adjunct to ceftazidime for the treatment of* S. pyogenes* and coexistence of oropharyngeal BLPB infections. Future studies should be investigated and confirmed in an animal test or humans. Also, the synergistic effect on blood and tissue would be evaluated and achieved.

## Figures and Tables

**Table 1:** Minimum inhibitory concentrations (MICs), fractional inhibitory concentration (FIC), and FIC index (FICI) determined by checkerboard assays of ceftazidime, amoxicillin, penicillin, nisin, luteolin, and quercetin either alone or in combination against *S. pyogenes* DMST 30653, 30654, and 30655.

Strains	MICs (*μ*g mL^−1^)						FIC^Ψ^ (*μ*g mL^−1^)		FICI^Σ^	
	Cef	Amo	Pen	Nis	Lut	Que	Cef + Lut	Cef + Que	Cef + Lut	Cef + Que
*S. pyogenes* DMST 30653	0.50^S^	N/D	N/D	1.0	128.0	128.0	0.12 + 16.0	0.12 + 4.0	0.37^SI^	0.27^SI^
*S. pyogenes* DMST 30654	0.50^S^	N/D	N/D	1.0	128.0	128.0	0.12 + 16.0	0.12 + 4.0	0.37^SI^	0.27^SI^
*S. pyogenes* DMST 30655	0.50^S^	N/D	N/D	1.0	128.0	128.0	0.12 + 16.0	0.12 + 4.0	0.37^SI^	0.27^SI^
*S. aureus* ATCC 29213^*∗*^	4.0^S^	0.5^S^	1.0^S^	1.0	N/D	N/D	N/D	N/D	N/D	N/D

^*∗*^ *S. aureus* ATCC 29213, amoxicillin, and penicillin were used as a positive control.

FIC^Ψ^ (*μ*g mL^−1^) value of Cef + Lut at 0.12 + 16.0 in each row below this column means MIC of ceftazidime at 0.12 plus luteolin at 16.0 *μ*g mL^−1^ in the combination.

FICI^Σ^ value of Cef + Lut at 0.37^SI^ in each row below this column means FIC of ceftazidime plus luteolin in combination was 0.37, which exhibited synergistic interaction.

S = susceptible; SI = synergistic interaction; N/D, not determined.

Cef = ceftazidime; Amo = amoxicillin; Pen = penicillin; Nis = nisin; Lut = luteolin; Que = quercetin.

The MICs are presented as the median values measured from three independent experiments; each experiment was performed in triplicate.
